# Upregulation of Cellular Bcl-2 by the KSHV Encoded RTA Promotes Virion Production

**DOI:** 10.1371/journal.pone.0023892

**Published:** 2011-08-25

**Authors:** Jianming Gao, Qiliang Cai, Jie Lu, Hem Chandra Jha, Erle S. Robertson

**Affiliations:** 1 Department of Microbiology and the Tumor Virology Program of the Abramson Cancer Center, Perelman School of Medicine at the University of Pennsylvania, Philadelphia, Pennsylvania, United States of America; 2 Department of Pathophysiology, Three Gorges University School of Medicine, Yichang, Hubei, China; Lisbon University, Portugal

## Abstract

Apoptosis of virus infected cells can restrict or dampen full blown virus propagation and this can serve as a protective mechanism against virus infection. Consequently, viruses can also delay programmed cell death by enhancing the expression of anti-apoptotic proteins. Human Bcl-2 is expressed on the surface of the mitochondrial membrane and functions as the regulator of the delicate balance between cell survival and apoptosis. In this report, we showed that the replication and transcription activator (RTA) encoded by KSHV ORF 50, a key regulator for KSHV reactivation from latent to lytic infection, upregulates the mRNA and protein levels of Bcl-2 in 293 cells, and TPA-induced KSHV-infected cells. Further analysis revealed that upregulation of the cellular Bcl-2 promoter by RTA is dose-dependent and acts through targeting of the CCN_9_GG motifs within the Bcl-2 promoter. The Bcl-2 P2 but not the P1 promoter is primarily responsive to RTA. The results of ChIP confirmed the direct interaction of RTA protein with the CCN_9_GG motifs. Knockdown of cellular Bcl-2 by lentivirus-delivered small hairpin RNA (shRNA) resulted in increased cell apoptosis and decreased virion production in KSHV-infected cells. These findings provide an insight into another mechanism by which KSHV utilizes the intrinsic apoptosis signaling pathways for prolonging the survival of lytically infected host cells to allow for maximum production of virus progeny.

## Introduction

Kaposi's Sarcoma-Associated Herpesvirus (KSHV), the etiological factor associated with Kaposi's Sarcoma is also known as human herpesvirus 8 (HHV 8). Several other malignancies such as primary effusion lymphoma, and multicentric Castleman's disease have also been known to associate with KSHV [1], [2], [3], [4]. KSHV belongs to the γ-herpesviruses family, the life cycle of which consists of two distinct phases, latent and lytic replication [5], [6], [7], [8]. During latency, the virus establishes persistent infection and only a small subset of genes such as ORF73, K12, ORF72, ORF71 and K15 are typically expressed [9], [10]. Under conditions of lytic reactivation such as hypoxia the virus spreads to new target cells and the viral genes are activated in cascade mode [11], [12], [13]. The switch to lytic reactivation is experimentally induced by a number of intracellular or extracellular motivators including the chemical agents 12-O-tetradecanoyl phorbol-13 acetate (TPA) and sodium butyrate [14], [15].

RTA, encoded by KSHV ORF 50, is an immediate early protein and functions as the critical regulator for the shift of KSHV life cycle from latency to lytic activation. Studies have previously shown that RTA is able to activate the transcription of many viral genes, including K1 [16], K2 [17], K3 [18], K5 [19], K8 [20], [21], K9 [22], K12 [23], K14[24], K15 [25], PAN RNAs [23], [26], [27], ORF35 [28], ORF49 [29], ORF50 itself [30], [31], K57 [32], [33], and K59 [34]. Furthermore, increased RTA expression which may be a result of expression from exogenous or endogenous sources is sufficient to disrupt viral latency and initiate KSHV lytic replication leading to the cascade reactivation of viral genes, host cell death and release of viral progeny.

Apoptosis is a major antiviral cellular response against viral infection. The B-cell leukemia/lymphoma 2 (Bcl-2) family of proteins controls the intrinsic mitochondrial pathway of cellular apoptosis [35], [36]. The Bcl-2 group of proteins is classified as pro- and anti- apoptotic members, all of which contain at least one highly conserved Bcl-2 homology (BH) domain [37]. The anti-apoptotic proteins, such as Bcl-2, Mcl-1, and Bcl-x_L_, share BH domains 1–4 [38]. The pro-apoptotic members include the Bax and the BH3-only families. The members of the bax family such as bax, bak share BH domains 1–3, and the BH3-only families have only the short BH3 motif respectively. This BH3 motif plays a central role in the killing action of molecules [37]. Moreover, Bcl-2 functions as one of the important regulators to maintain the delicate balance between cell survival and apoptosis. The human Bcl-2 gene is overexpressed in numerous human cancers, including B- and T- cell lymphomas, cervical, lung, breast, prostate and colorectal cancers [39], [40], [41], [42], [43], [44]. Its overexpression in tumor cells not only functions as an apoptosis inhibitor, but also results in resistance to chemotherapy or radiotherapy-induced apoptosis [45], [46]. Thus, because of its function in anti-apoptosis, the Bcl-2 gene has become a strong potential target in development of anticancer therapies [47], [48], [49].

Bcl-2 protein levels can be regulated transcriptionally and post transcriptionally. Furthermore, the human Bcl-2 gene contains both the P1 and P2 promoters [47], [49]. P1, the predominant promoter, is located 1.3- to 1.5-kbp upstream of the translation start site. The P1 promoter is mainly GC rich, with a number of transcription initiation sites and includes seven consensus binding sites for the Sp1 transcription factor. However, the P2 promoter has canonical CAAT as well as TATA boxes [47], [49]. Some transcriptional factors such as CREBP (cAMP responsive element binding protein), and NFκB are known to be positive regulators of Bcl-2 transcription, and a few negative regulatory sites have also been described, including WT1, p53 binding sites [47]. Studies have shown that G-quadruplex structure upstream of the P1 promoter plays a role in transcription regulation [48], [49]. In addition, Bcl-2 may also be modified post-translationally by phosphorylation which occurs at mitogen-activated protein kinase (MAPK) sites which can trigger proteasome-dependent degradation and ubiquitination [50]. Caspase-dependent cleavage can also occur leading to loss of its anti-apoptotic activity [50].

We now show that RTA can transactivate the cellular Bcl-2 promoter in a dose-dependent manner. RTA upregulates Bcl-2 through targeting of CCN_9_GG-like RTA responsive elements (RREs) within the promoter and that the P2 promoter is important for RTA-mediated transcriptional regulation of cellular Bcl-2. These findings provide insights into new mechanisms by which RTA transactivates cellular Bcl-2 and so contributes to enhanced efficiency of KSHV lytic replication.

## Results

### Bcl-2 transcipts and protein levels are elevated in cells transiently expressing RTA

**Figure 1:**
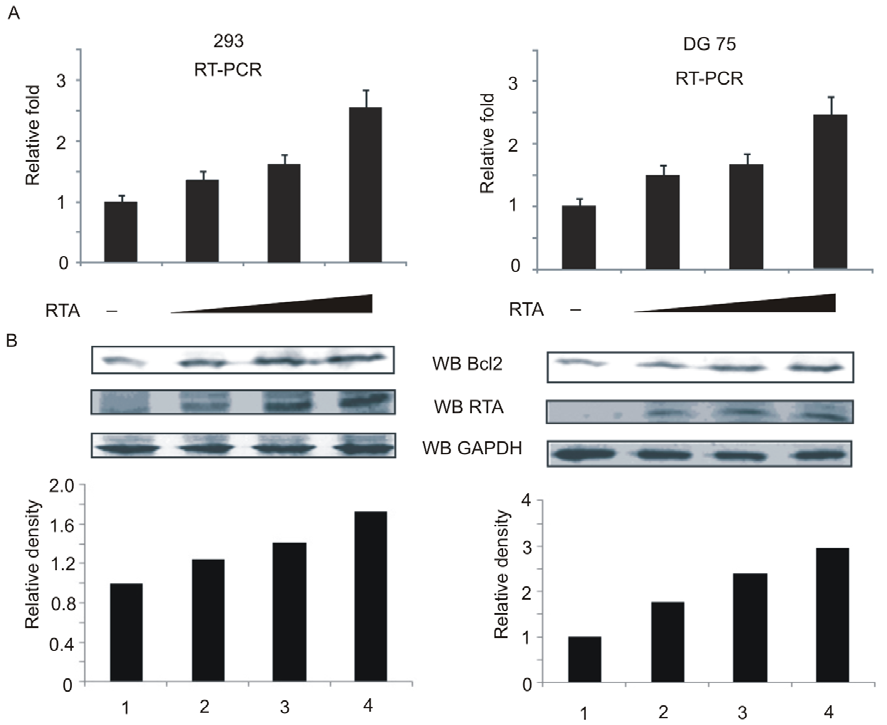
Bcl-2 is upregulated in HEK 293 and DG 75 cells tranfected with RTA. (A) Quantification of Bcl-2 transcripts by RT-PCR. Total RNA was ectracted from 10 million HEK 293 and DG 75 cells tranfected with increasing amount of RTA plasmid (0, 5, 10, 15 µg). A total of 2 µg of RNA was used to synthesize cDNA. Real-time PCR was performed by using a power SYBR green PCR Master Mix kit with GAPDH as control. Standard deviations were illustrated by error bars. (B) Western blot (WB) analysis of endogenous Bcl-2 in the RTA-expressing HEK 293. GAPDH was used for internal control. Quantification of the relative densities of Bcl-2 was plotted below the blots.

Bcl-2 mRNA transcripts and protein levels were examined to specifically detect the RTA-mediated cellular Bcl-2 upregulation (Fig. 1). HEK 293 and DG75 cells were transiently transfected with increasing amounts of RTA expression plasmid. Total RNAs were isolated from cells for use in RT-PCR assay. The results of the RT-PCR analysis showed that RTA clearly enhanced the Bcl-2 transcript levels in a dose- dependent manner. Furthermore, results of Western blot analyses also supported increased upregulation of Bcl-2 protein in a dose-dependent manner (Fig. 1A, B). Therefore, expression of RTA directly affects the transcription of Bcl-2 and upregulates Bcl-2 protein levels.

### Bcl-2 expression is upregulated in TPA-induced KSHV-infected BC3 and BCBL1 cells

**Figure 2:**
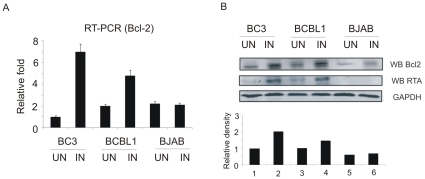
Bcl-2 is upregulated in TPA and butyrate induced BC3, BCBL1 cells. (A) Quantification of Bcl-2 transcripts by RT-PCR. (B) Western blot analysis of endogenous Bcl-2 in induced and uninduced BC3, BCBL1, BJAB cells.

KSHV lytic replication can be initiated by chemical inducers, like TPA and sodium butyrate [51]. Being an immediate early protein, expression of RTA can be observed at the first hour after TPA treatment [55], [56], [57]. To determine the changes in Bcl-2 level in KSHV induced cells, KSHV was induced from BC3 and BCBL1 cells as mentioned above and cells were harvested for analysis. The results from our RT-PCR and Western blot analyses showed that Bcl-2 expression were strongly upregulated in TPA and butyrate induced KSHV-positive B cells (Fig. 2A, B). In contrast, there was no background RTA expression in uninduced cells subjected to the same analysis, and as expected in the KSHV-negative BJAB cell there was no RTA expression after induction. Moreover, the expression of Bcl-2 mRNA and protein in BJAB cells when treated with TPA had little or no obvious effect when uninduced cells were compared to the induced cells suggesting that the upregulation of Bcl-2 is due to the presence of KSHV and the viral induced factor RTA (Fig. 2).

### The levels of Bcl-2 mRNA is enhanced in RTA-expressing HEK 293 cells and in KSHV-infected B cells induced for lytic replication

**Figure 3:**
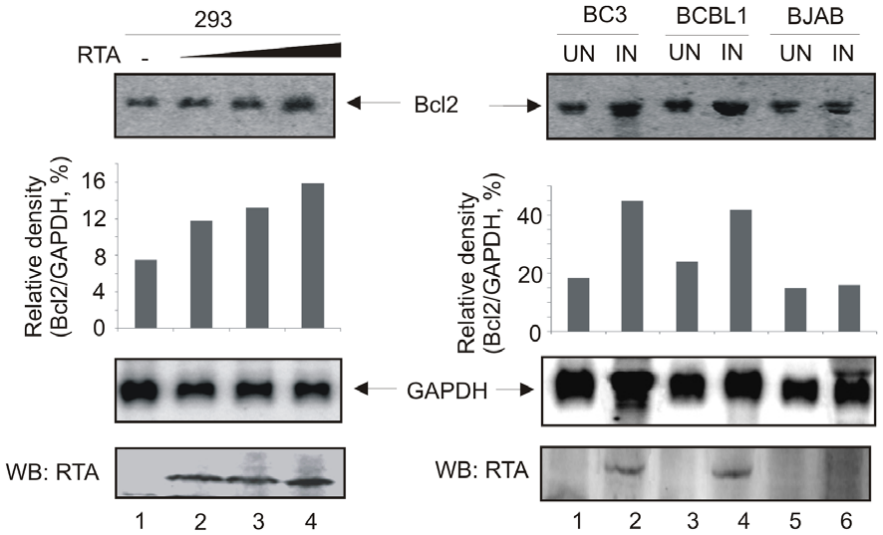
Northern blot analyses of Bcl-2 RNA. Total RNA was extracted from each cell (HEK 293, RTA-expressing HEK 293, induced and uninduced BC3, BCBL1, BJAB cells). Blots were first probed against Bcl-2 mRNA, then reprobed with a GAPDH probe. Density of Bcl-2 signal divided by that of GAPDH control was used to quantify the levels of Bcl-2 mRNA expression. Western blot analysis was used to detect expression of RTA transcript to show the dose effect.

In addition to RT-PCR analysis, we wanted to corroborate our results and so performed Northern blot analysis to verify Bcl-2-specific mRNA in RTA expressing HEK 293 cells and in induced KSHV-infected B cells by using a Bcl-2-specific DNA probe. Results showed that the levels of total Bcl-2 mRNA were strongly increased in a dose-dependent manner in HEK 293 cells transfected with the RTA expression plasmid. Additionally, total Bcl-2 mRNA expression in TPA induced BC3 and BCBL1 cells was also strongly enhanced compared to uninduced cells with no evidence of change in KSHV negative BJAB cells. The same blots were re-probed with a GAPDH specific probe as control. The density of Bcl-2 signal was normalized against that of the GAPDH control to quantify the levels of Bcl-2 mRNA expression and was presented as a histogram for relative density levels (Fig. 3).

### RTA activates the Bcl-2 gene promoter in a dose-dependent manner

**Figure 4:**
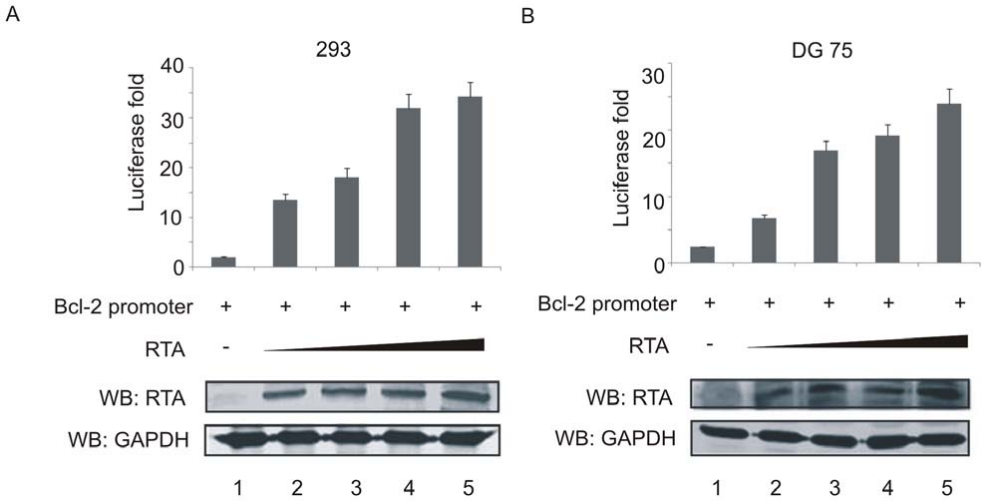
RTA transactivated the Bcl-2 promoter in a dose-dependent manner. (A, B) Ten million HEK 293 cells or DG75 cells were transfected with 10 µg of PGL3-Bcl-2, and 0, 5, 10, 15, or 20 µg pcDNA-RTA. Cell lysates were prepared at 24 h posttransfection for the luciferase assay. Data from three independent groups are exemplified with mean and standard deviation to show the fold activation by comparison of the promoter activity in the presence of RTA with the value of pGL3B alone. Western blot was used to confirm the transfection efficiency with GAPDH served as control for equal protein loading.

Since TPA-induced KSHV-infected cells and RTA expressing cells showed higher levels of Bcl-2 mRNA and protein expression, we further explored the molecular mechanism of Bcl-2 promoter up-regulation by RTA. To analyze the role of RTA on regulating the Bcl-2 promoter, a fixed amount of full length Bcl-2 promoter luciferase construct (pGL3-Bcl-2) [47], , was cotransfected with increasing amounts of pcDNA-RTA into HEK 293 and DG75 cells. After 24 h, the cells were harvested for luciferase reporter assay. The reporter assays showed that RTA can indeed activate the Bcl-2 promoter by approximately 12 to 35-fold compared to that of the vector alone in HEK 293 cells as well as 5 to 25-fold in DG75 cells (Fig. 4 A and B). These results suggest that RTA can up-regulate Bcl-2 expression through regulation of cis-acting elements in the promoter.

### The P2 promoter and the CCN_9_GG domains both contribute to Bcl-2 promoter activation by RTA

**Figure 5:**
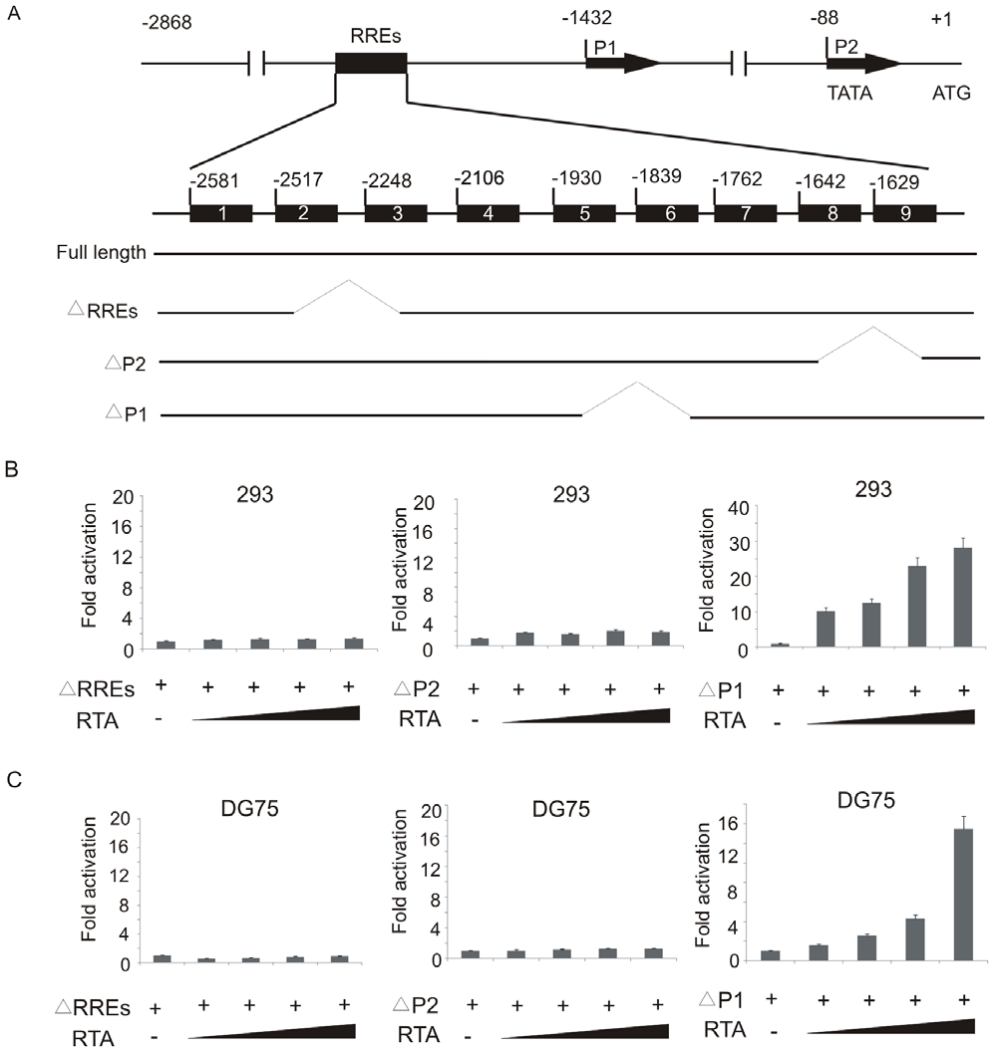
The CCN_9_GG-like KSHV RTA responsive element (RRE) and P2 promoter region are indispensable for activation of Bcl-2 transcription by RTA. (A) Illustration of the wild type and mutant Bcl-2 promoters. Nine CCN_9_GG motifs in Bcl-2 promoter are shown. Numbers represent the nucleotide positions upstream of the initiating ATG. (B, C) Ten million HEK 293 cells or DG75 cells were transfected with 10 µg of indicated mutant Bcl-2 promoter reporter plasmids, and 5, 10, 15, or 20 µg pcDNA-RTA. Cell lysates were prepared at 24 h posttransfection for the luciferase assay. The deletion of P1 promoter did not result in a major decrease in promoter activity and ever resulted in an increase in promoter activity in HEK 293 cells. However, deletion of either RREs or P2 promoter resulted in complete loss of Bcl-2 promoter activity in the presence of RTA.

To identify the potential motifs indispensable for the upregulation of cellular Bcl-2 by RTA, a series of truncated promoter luciferase contructs were generated and tested by reporter analysis in both HEK 293 and DG75, as shown in Fig. 5 A to C.

The two promoters P1 and P2 [47], [49] and 9 CCN_9_GG-like RTA-responsive elements (RREs) are located within the full length Bcl-2 promoter (Fig. 5A). The CCN_9_GG-like sequences within the Bcl-2 promoter are similar to one of the RREs identified in the KSHV ORF57 promoter [58]. To identify which of these motifs play a critical role in regulating transcription of the Bcl-2 promoter by RTA, three truncations of the promoter named pGL3-ΔRREs, pGL3-ΔP1, pGL3-ΔP2 were generated to delete the RREs, the P1 and the P2 promoters, respectively. These promoter constructs were transfected into HEK 293 and DG75 cell lines with different amounts of RTA plasmids. As shown in the Fig. 5B and C, the deletion of P1 promoter did not result in change in promoter activity in HEK 293 cells (Fig. 5B). In contrast, deletion of P2 promoter as well as the deletion of the 9 CCN_9_GG-like RREs resulted in a complete shutdown of the promoter activity with an almost negligible change in activity when compared to promoter alone without RTA expression. Our data strongly indicated that the P2 promoter region and the CCN_9_GG domains were indispensable for RTA-mediated upregulation of Bcl-2.

**Figure 6:**
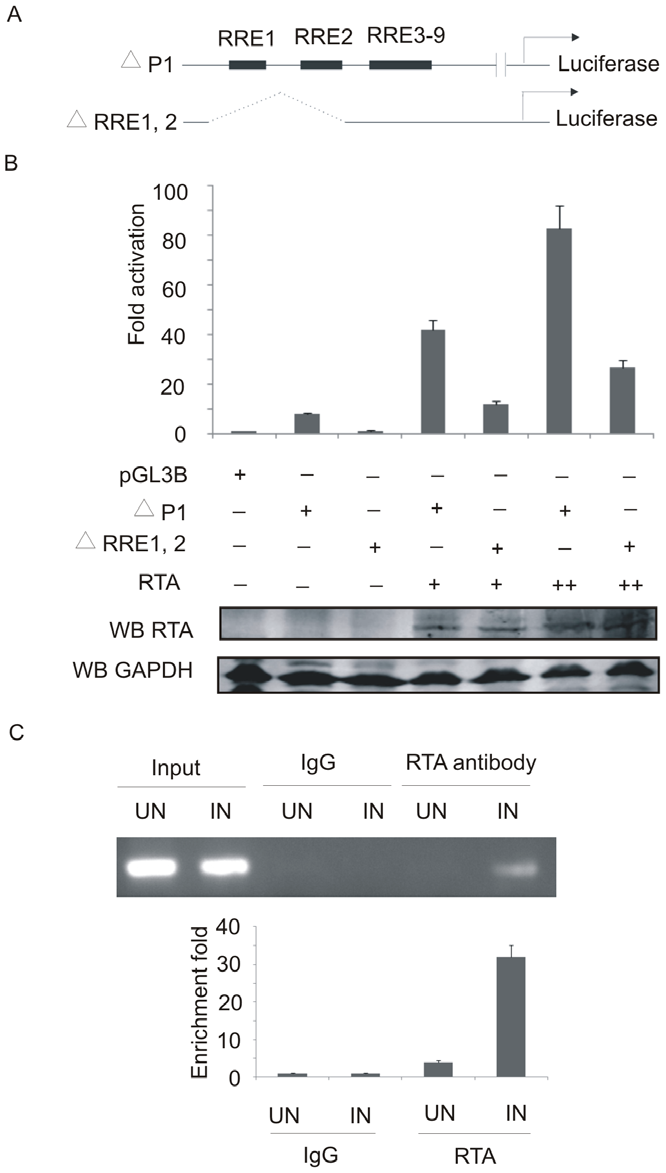
The CCN_9_GG motifs contribute to Bcl-2 promoter activation by RTA. (A) Scheme showing pGL3-ΔRRE1,2 and pGL3-ΔP1 construct used in this study. (B) Fix amounts (10 µg) of indicated mutant Bcl-2 promoter luciferase constructs were transfected or cotransfected into ten million HEK 293 cells with 10, 20 µg of pcDNA-RTA respectively. Cell lysates were prepared and the luciferase assay was carried out as above mentioned in Fig. 4 legend. Deletion of the first and second CCN_9_GG motifs led to a significant decrease in RTA-mediated Bcl-2 promoter activity. (C) A ChIP assay was carried out using induced or uninduced BCBL1 cells. RTA-DNA complexes were specifically precipitated with or without RTA antibody. The precipitated DNA was recovered by PCR with primers covering RRE 1 and 2 (upper panel). Real-time PCR was performed to quantify the immunoprecipitated promoter DNA.

To confirm the role of the CCN_9_GG motifs in Bcl-2 regulation, we performed reporter assays by using the pGL3-ΔRRE1,2 luciferase construct in which the first and second CCN_9_GG motifs were deleted (Fig. 6A). We found that mutation of the first and second CCN_9_GG RRE motifs in the Bcl-2 promoter dramatically diminished promoter activity compared with that of the pGL3-ΔP1 alone (Fig. 6B). First of all without RTA there was little or no activity comparable to basic luciferase vector alone. However, with the RTA expression construct the results showed about a 10 fold increase with the ΔRRE1,2, but a decrease of approximately 3 times compared to the ΔP1 reporter which contained all the RREs (Fig. 6B). This suggests that the CCN_9_GG motifs within the Bcl-2 definitely play a critical role in RTA-mediated regulation of this promoter. To show direct interaction of RTA protein with the CCN_9_GG motifs, CHIP was carried out using induced and uninduced BCBL1 cells. The presence of the first and second CCN_9_GG motifs within the immunoprecipitated complex was confirmed by PCR amplification. A clear band was detected in RTA antibody precipitated induced nuclei DNA, whereas there is no signal in RTA antibody precipitated uninduced nuclei DNA, and the two IgG negative controls failed to recover any PCR product (Fig. 6C, upper panel). Real-time PCR was also used to quantify the precipitated DNA. The amount of RTA antibody precipitated induced nuclei DNA was about 8 to 32 times higher when compared with that of the control groups (Fig. 6C, bottom panel).

### Bcl-2 expression is important for prolonged survival of lytically infected host cells and contributes to enhanced production of virus progeny

**Figure 7:**
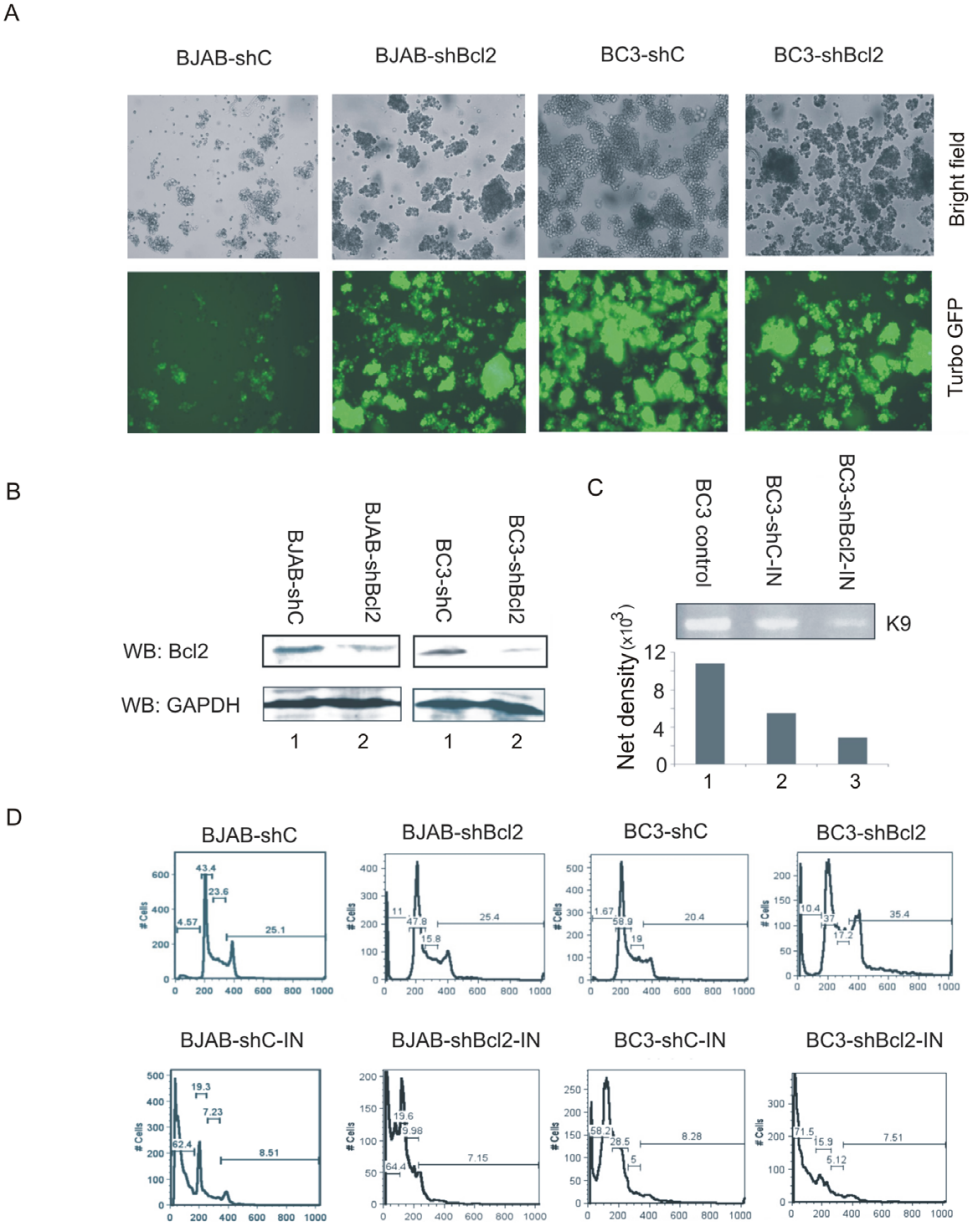
RTA-mediated Bcl-2 upregulation is important for prolonging the survival of lytically infected host cells and to enhance production of virus progeny. (A) Bcl-2 knockdown cells and control cells with GFP expression were monitored by fluorescence microscopy showing successful lentivius infection. (B) Western blots (WB) showing expression of Bcl-2 in BJAB-shC, BJAB-shBcl-2, BC3-shC, and BC3-shBcl-2 cells. GAPDH served as control. (C) Supernatants of RTA and butyrate-induced BC3-shC, and BC3-shBcl2 cells were prepared after induction 48 h for PCR to analyze the production of KSHV progeny. KSHV genomic DNA isolated from BC3 cells served as control. (D) Different cellular apoptosis pattern in induced and uninduced BJAB-shBcl2 and BC3-shBcl2 cell lines. The first sub-G1 peak represents apoptotic cell.

On the basis of the results we observed above, we hypothesized that RTA could take advantage of the Bcl-2 pathway to promote cell survival which may result in enhanced virus progeny production. Hence, we wanted to determine whether upregulation of cellular Bcl-2 by RTA may in fact affect cellular apoptosis. We constructed stable Bcl-2 knockdown cells (BC3-shBcl2, BJAB-shBcl2) and their control cells (BC3-shC,B JAB-shC) by lentivirus infection and puromycin selection (Fig. 7A). The expression of Bcl-2 in the Bcl-2 knockdown and the control cell lines were monitored by Western blot. Bcl-2 protein level was considerably decreased in both BJAB and BC3 cells transduced with the shBcl-2 lentivirus (Fig. 7B). To quantitate the KSHV virus progeny production, the virus supernatant of induced BC3-shC or BC3-shBcl2 cells was prepared at 48 h postinduction, and used as a template for PCR analysis of the KSHV ORF9. Our data indicated that Bcl-2 knockdown reduced the production of virus particles as determined by detection of the quantified product of the K9 gene amplified by PCR (Fig. 7C). This was about 2-fold less than the progeny production in the control cell line. These results indicate that RTA-mediated enhanced expression of Bcl-2 contributes to virus progeny production during lytic replication. The two cell lines examined had a distinctly different pattern of apoptosis when treated with TPA and butyrate. Under induction, the BJAB cell line which is negative for KSHV resulted in similar levels of apoptosis which was about 62–64% when the BJAB-shC cells were compared to the BJAB-shBcl2 cells (Fig. 7D). Importantly, the results from the KSHV-infected BC3 cells showed a clear increase in the level of apoptosis when comparing the BC3-shC cells to the BC3-shBcl2 cells (p<0.05) (Fig. 7D). These findings provide convincing evidence supporting the hypothesis that RTA can upregulate Bcl-2 expression and have a direct role in enhancing cell survival and thus an increase in virus progeny production.

## Discussion

Similar to other large DNA herpesviruses, KSHV has two major life cycles referred to as the latent and lytic replication cycles. Latency exists in most of the KSHV infected cells which harbor the viral episomes [59], [60], but a minority of cells do undergo the full lytic program to sustain KS pathological changes [61], [62]. KSHV viral genes are successively activated in the lytic replication phase which can be triggered by RTA [53]. RTA regulates the KSHV lytic phase to reactivate the virus from latency and so initiate replication [55], [56], [57]. Previous studies showed that RTA transactivates the transcription of its downstream gene in two ways, one is through binding RREs, and another is by interacting with cellular transcription factors, such as K-RBP [63], [64], CEBP-α [33], [65], Oct-1 [30], and RBP-Jκ [24].

Apoptosis of infected cells can restrict or dampen full blown virus replication and this can serve as a protective defense mechanism against virus infection. Consequently, viruses can also delay programmed cell death by enhancing the expression of antiapoptotic proteins [66], [67], [68]. The ability to suppress apoptosis may be essential to drive the enhanced survival of KSHV-positive cells so that the virus may replicate and spread, thus resulting in persistence of the virus in the host. KSHV can employ a number of molecular strategies which are related to induction of cell transformation, regulation of cell proliferation, and resistance of apoptosis. Therefore, there is a high probability that RTA contributes to virus lytic replication through the activation of cellular Bcl-2 expression very early during infection.

Human Bcl-2 is expressed on the surface of the mitochondrial membrane and functions as a major regulator of the delicate balance between cell survival and apoptosis [47], [49]. In this study, we now present evidence for the interplay between RTA and Bcl-2. Both Bcl-2 mRNA and protein levels were upregulated in RTA-expressing human cells and in TPA-induced KSHV-infected cells. Furthermore, we demonstrated that RTA may in fact directly activate the Bcl-2 gene promoter. As expected, results of the reporter assay further established that activation of Bcl-2 by RTA was dose-dependent, and that the effect was not cell type specific. Thus, these experiments have now convincingly established an association between RTA expression and Bcl-2 upregulation.

To determine the mechanisms by which RTA activates Bcl-2, we identified potential responsive elements within the Bcl-2 promoter. Interestingly, this promoter contains nine CCN_9_GG-like motifs and these CCN_9_GG-like motifs were recently identified KSHV RRE present in five gene promoters of KSHV [58] . Both experiments in vitro and in vivo have shown that this RRE was able to react with RTA so as to regulate the activation of RTA [58]. This provided the likelihood that a similar strategy may be used in upregulation of Bcl-2 by RTA. Our data from the reporter assays with a series of truncated promoters demonstrated that these CCN_9_GG-like motifs in fact do have an effect on transactivation of Bcl-2 by RTA. Deletion of the nine CCN_9_GG motifs completely shut down the Bcl-2 promoter activity. Additionally, deletion of the first and second CCN_9_GG motifs also led to a significant decrease in RTA-mediated Bcl-2 promoter activity. Furthermore, the results of ChIP confirmed the direct interaction of RTA protein with RREs within the Bcl2 promoter. This confirms our hypothesis that the CCN_9_GG motifs are important and likely to be critical for transactivation of Bcl2 by RTA. Interestingly, a highly consistent sequence, GNCCN_9_GGNG, with an identification as EBV RRE has been reported [69]. Another previous study on the EBV RTA-responsive promoters pointed out that the differential responsiveness of the CCN_9_GG motifs to RTA transcription and KSHV reactivation may result from a variation in the N_9_ sequences [70]. The KSHV five gene promoters containing the consensus sequence CCN_9_GG also contained variations in the N_9_ sequences [58]. The PAN-RRE had stronger interactivity with RTA than the K2- and K12-RREs [23]. A few cellular factors, as mentioned above, can clearly interact with RTA [53]. Their cis-acting binding domains are possibly in the vicinity of the CCN_9_GG sequence [58]. Thus, RTA can either bind to the CCN_9_GG motif or these cellular proteins bound to their cis-acting elements which may result in optimal activation of the promoter. In addition, our data also showed that the P2 promoter is indispensable for RTA-mediated transactivation of Bcl-2. The pGL3-ΔP1 construct had no detectable reduction of luciferase activity compared with the full length Bcl-2 promoter, whereas mutation of P2 promoter region was able to completely shut down the promoter activity. The P2 promoter contains both a CCAAT and a TATA box and an octamer motif [71]. However, the P1 region contains a TATA-less GC-rich promoter with seven binding sites for the Sp1 transcription factor [71]. How is it that the P2 promoter has a preference for Bcl-2 transactivation by RTA? We cannot however exclude the possibility that the TATA box and/or the octamer motif within the P2 promoter are indispensable for RTA-mediated upregulation of Bcl-2. These studies are yet to be done and will hopefully provide a deeper understanding of the mechanism.

Depletion of Bcl-2 clearly increases the level of cellular apoptosis, and decreases virus progeny production in induced BC3-shBcl2 cells compared with that of the BC3-shC cells. This provides strong evidence that upregulation of cellular Bcl-2 by RTA can definitely contribute to KSHV lytic replication. The utilization of the Bcl-2 pathway by RTA would be a responsible strategy for prolonging the survival of lytically infected cells and thus allow enhanced production of virus progeny.

**Figure 8:**
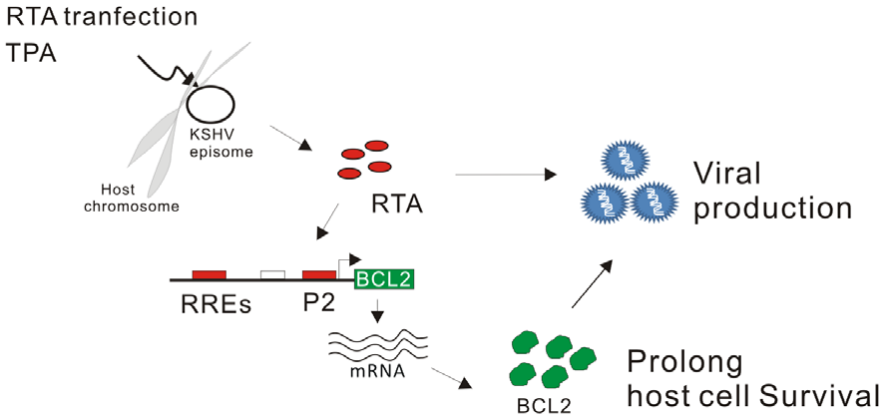
Hypothetical model showing RTA mediated upregulation of cellular Bcl-2. P2 promoter and the CCN9GG-like RREs are indispensable for upregulation of Bcl-2 by RTA. Enhanced expression of Bcl-2 prolongs cell survival and virus progeny production. RRE, RTA responsive element.

In summary, our study which investigated the interaction between KSHV RTA and cellular Bcl-2 showed that RTA can transactivate Bcl-2 transcription through targeting of the RRE CCN_9_GG motifs within the Bcl-2 promoter. We also demonstrated that the P2 promoter region instead of the P1 promoter region was highly responsive to RTA. The results of ChIP confirmed the direct interaction of RTA protein with the CCN_9_GG motifs. Knockdown of cellular Bcl-2 by lentivirus-delivered small hairpin RNA (shRNA) resulted in increased cell apoptosis and decreased virion production in KSHV-infected cells. RTA can upregulate Bcl-2-mediated block to cellular apoptosis, and so prolong cell survival leading a further increase in production of virus progeny (Fig. 8). These findings provide an insight into the molecular mechanism by which KSHV interferes with the intrinsic apoptosis signaling pathways to promote viral lytic replication.

## Materials and Methods

### Constructs

Plasmid pcDNA-RTA is an expression vector which encodes the full-length RTA. The Bcl-2 gene full-length promoter reporter plasmid, pGL3-Bcl-2 was a kind gift from Véronique Bourgarel-Rey (Aix-Marseille Université, Marseille Cedex 05, France). The truncated reporter plasmids pGL3-ΔRREs, pGL3-ΔP1, pGL3-ΔP2 and pGL3-ΔRRE1,2 were obtained by PCR-based site mutagenesis (Primers see Table 1). Plasmids pGL3-ΔRREs and pGL3-ΔP2 were generated by inserting PCR amplicons of the Bcl-2 promoter (nucleotide −1563∼+1 for ΔRREs; −2744∼−680 for ΔP2) into the SmaI/HindIII sites of pGL3 basic vector. For construct pGL3-ΔP1, first inserting the promoter fragment (−346∼+1) near the start codon into the MluI/HindIII sites of the pGL3 basic vector (pGL3-P2), then followed by inserting another PCR fragment (−2867∼−1545) into the KpnI/MluI sites of pGL3-P2. Construct pGL3-ΔRRE1,2 was derived by deletion of the first and second CCN_9_GG motifs within pGL3-ΔP1.

**Table 1:** Primers for PCR amplification and analysis.

Primer	Squence*	Note
Bcl-2-F1	5′-GGTGAACTGGGGGAGGATTGT-3′	Real-time PCR
Bcl-2-R1	5′-CTTCAGAGACAGCCAGGAGAA-3′	
GAPDH-F1	5′-TGCACCACCAACTGCTTAG-3′	Real-time PCR
GAPDH-R1	5′-GATGCAGGGATGATGTTC-3′	
Bcl-2-F2	5′-TCTGCGAAGAACCTTGTGTG-3′	Northern probe
Bcl-2-R2	5′-CTCCACCAGTGTTCCCATCT-3′	
GAPDH-F2	5′- TGGAAGGACTCATGACCACA-3′	Northern probe
GAPDH-R2	5′- AGGGGTCTACATGGCAACTG-3′	
Bcl-2Δ1	5′-CCCAAGCTTCCTTCCCAGAGGAAAAGC-3′	ΔRREs clone
Bcl-2Δ2	5′- TGAATGAACCGTGTGACGTT -3′	
Bcl-2Δ3	5′-TGCTAGGGGCTATTCATGCT-3′	ΔP2 clone
Bcl-2Δ4	5′-CCCAAGCTTCCAGATCGATTCCCAGAC-3′	
Bcl-2Δ5	5′-CCCGGTACCTCGAGCCCTATTAAGTAAG-3′	ΔP1 clone
Bcl-2Δ6	5′-CCCACGCGTAACGTCACACGGTTCATTCA-3′	
Bcl-2Δ7	5′-CCCACGCGTGTGTACAGGGAAACGCACCT-3′	
Bcl-2Δ8 (Δ1)	5′-CCCAAGCTTCCTTCCCAGAGGAAAAGC-3′	
Bcl-2Δ9	5′-CCCGGTACCGCCCTATTAAGTAAGCCGCTGT-3′	ΔRRE1,2 clone
Bcl-2Δ10	5′-CCCACGCGTGGTGGGCTCCTAAAGGTCTC-3′	
Bcl-2Δ11	5′-CCCACGCGTAGGGACAGAGGGAAAACATTG-3′	
Bcl-2Δ12 (Δ1)	5′-CCCAAGCTTCCTTCCCAGAGGAAAAGC-3′	
K9-F	5′-CCAGGAAAAGAGCAGTCAGG-3′	PCR for virus progeny
K9-R	5′-CGCGTTGAGCACTTGAATTA-3′	
ChIP-F	5′-GCCAACTAGGATTGGCTACG-3′	ChIP assay
ChIP-R	5′-CGTGTCCACCTGAACACCTA-3′	

* Restriction sites are underlined.

### Antibodies and cell cultures

RTA antibody was generated from a hybridoma (Lan et al. Institute Pasteur, Shanghai, China). The Bcl-2 mouse monoclonal antibody is a product of Santa Cruz Biotechnology, Inc (Santa Cruz, CA). The GAPDH mouse antibody was obtained from Novus Biologicals, LLC (Littleton, CO).

Human embryonic kidney fibroblast 293 was obtained from Jon Aster (Brigham and Women's Hospital, Boston, MA). BJAB is a KSHV and EBV negative B cell line and was provided by Elliott Kieff (Harvard Medical School, Boston, MA). DG75 is a KSHV negative B cell line. BC3 and BCBL1 are KSHV-positive body cavity-based lymphoma-derived cell lines obtained from the NIH AIDS Research and Reference Reagent Program [51]. DG75 were obtained from the American Type Culture Collection. Cells were cultured as described before [47], [52].

### Transfection and Induction of KSHV

HEK 293 and DG75 cells were transfected by using a Bio-Rad Gene Pulser II electroporator. Experimental method for transfection by electroporation was previously described [53]. BC3, BCBL1 and BC3-shBcl-2 cells were induced with 20 ng of TPA/ml and 1.5 mM butyrate [51]. BJAB, BJAB-shC, BC3-shC cells were used as control. All cells were harvested for experimental use at 48 h postinduction.

### Western blot

After treatment, the cells were harvested and lysed in a RIPA buffer. The protein concentration of the lysates was measured by Bradford assay. Samples were subjected to sodium dodecyl sulfate-polyacrylamide gel electrophoresis (SDS-PAGE) and then transferred to polyvinylidene difluoride (PVDF) membranes. Western boltting was performed using anti-Bcl2, anti-RTA, or anti-GAPDH antibody and goat anti-mouse IgG secondary antibody conjugated to IR dye 800 (Rockland Immunochemicals, Inc., Gilbertsville, PA). After the membranes were washed, the signals were scanned with Odyssey infrared image (LI-COR, Inc., Lincoln, NE).

### Quantitative real-time PCR analysis

Total RNA extraction from cells (HEK 293, RTA-transfected HEK 293, DG75, RTA-transfected DG75, induced and un-induced BC3, BCBL1, BJAB cells), generation of cDNA, preparation of reaction mixture and RT-PCR amplification condition were described previously with minor modifications [47], [52]. The primer sequences of Bcl-2 and GAPDH genes used for the real-time PCR are shown in Table 1 [47], [52]. Data analyses were carried out by using a StepOnePlus RT-PCR system (Applied Biosystems, INC., Foster City, CA) with each sample tested in triplicates.

### Northern blot analysis

A human Bcl-2 gene DNA probe of 539 bp and glyceraldehyde-3-phosphate dehydrogenase (GAPDH) probe of 628 bp were generated by PCR using HEK 293 cells cDNA as template. Primer 3 software was used to design the primers (listed in Table 1). Both DNA probes were confirmed by sequencing (sequencing core, Upenn). The DNA probes were radiolabeled by random priming method with NEB #1500 L kit (New England Biolabs, Ipswich, MA). Ten microgram of total RNA from each cell (HEK 293, RTA-transfected HEK 293, induced or uninduced BC3, BCBL1, BJAB cells) was resolved by formaldehyde-agarose gel electrophoresis and transferred to a 0.22 µm pore nitrocellulose membrane. After prehybridization, the membrane was hybridized successively with ^32^P-radiolabeled DNA probes, followed by washes twice with SSC 2× buffer (0.3 M NaCl, 30 mM sodium citrate, pH 7.0, 0.1% SDS) for 5 min at room temperature, then with SSC 0.5× buffer, 0.1% SDS for 10 min at 60°C. The autoradiographic images were analyzed by using a PhosphorImager with supplementary ImageQuant software (Molecular Dynamics Inc., Sunnyvale, CA).

### Luciferase assay

Ten million HEK 293 and DG75 cells were cotransfected with 10 µg of the reporter plasmids and increasing amounts of RTA plasmids respectively. Cell lysates were prepared at 24 h posttransfection. Twenty five microliters of luciferase assay substrate was mixed with 40 µl cell lysate, and a LMaxII384 luminometer (Molecular Devices, Inc., Sunnyvale, CA) was used to measure luminescence level. The results showed the mean and standard deviation (SD) of the data of three independent experiments. Western blot analysis was used to confirm the expression of the proteins.

### Chromatin immunoprecipitation (ChIP)

At 48 h post-induction, BCBL1 cells were fixed by adding 37% formaldehyde to a final concentration of 1% for 10 min. The cross-linking reaction was stopped by adding glycine to a final concentration of 0.125 M for 5 min. Cells were washed three times with 1× ice-cold PBS and resuspended in cell lysis buffer [5 Mm PIPES (pH 8.0), 85 mM KCl, 0.5% NP-40] containing 1 mM PMSF and 1 µg/ml protease inhibitors for 10 min at 4°C. After centrifugation, the cell pellets were resuspended in nuclear lysis buffer [50 mM Tris/HCl (pH 8.1), 10 mM EDTA, 1% SDS] and incubated for 10 min at 4°C. The resulting solution was diluted 5-fold with dilution buffer [16.7 mM Tris/HCl (pH 8.1), 167 mM NaCl, 1.2 mM EDTA, 0.01% SDS, 1.1% Triton X-100, 1 mM PMSF, 1 µg/ml protease inhibitors] and sonicated. After centrifugation, the supernatant was pre-cleared with mouse IgG and immobilized Protein A. Immunoprecipitation was performed by incubation at 4°C with anti-RTA antibody or mouse IgG antibody. Immune complexes were collected by a further incubation with immobilized Protein A for 1–2 h at 4°C. The beads were washed four times with IP buffer [25 mM Tris/HCl (pH 7.2), 150 mM NaCl] and resuspended in elution buffer (1% SDS/0.1 M NaHCO_3_). After centrifugation, the supernatant was neutralized with 1 M Tris/HCl (pH 7.2). RNase A and NaCl were added, followed by incubation at 65°C overnight to reverse the cross-linked DNA–protein complex. Proteinase K was added, followed by incubation at 55°C for 1–2 h. DNA extraction was carried out using phenol: chloroform and analysed by PCR using specific primers covering RRE1 and RRE 2 in the full length reporter vector. The DNA was quantified by SYBR green real-time PCR. For calculation of the relative DNA amount from quantitative real-time PCR, the Ct (threshold cycle) value of antibody-precipitated sample was normalized by the Ct value of the input, and the normalized Ct value of induced and uninduced groups were compared.

### RNA interference lentiviral constructs and transduction

Bcl-2 small hairpin RNA (shBcl-2) and control shRNA (shC) were synthesized by Sigma (St. Louis, MO) as previously described [54]. A shRNA lentiviral vector expressing Bcl-2 small hairpin, pGIPZ, was a product of Open Biosystems, Inc (Huntsville, AL). Protocols for lentiviruses infection were previously described [47], [52]. In our experiments, BC3-shBcl2, BJAB-shBcl2 and their corresponding control cell lines, BC3-shC and BJAB-shC were generated for further analysis.

### PCR amplification of viral progeny DNA

To investigate whether Bcl-2 upregulation can increase the production of viral progeny, the KSHV encoded ORF9 was amplified by PCR using viral DNA in TPA and butyrate induced BC3-shC and BC3-shBcl2 cells. Cells were induced and further incubated for 2 days at 37°C under 5% CO_2_. The supernatant was then collected and passed through a 0.45-µm-pore-size filter, and viral particles were spun down at 23,500 rpm for 20 min. Intact cells were discarded to prevent possible lysis and contamination from cellular viral DNA. The pellet was resuspended in 50 µl of 0.2× PBS, heated to 95°C for 15 min, and switched to 56°C for 1 h with proteinase K treatment (10 mg/ml). The enzyme was then destroyed by treatment at 95°C for 30 min. A 10-µl portion of virus lysate was used for PCR amplification of the KSHV-specific region of ORF9. The primers for amplification of ORF9 are listed in Table 1. KSHV genomic DNA isolated from BC3 cells served as control. The net density of the PCR product bands was quantitated using Kodak 1D 3.6 software (Eastman Kodak Company, Rochester, NY).

### Apoptosis assay

Cellular apoptosis patterns were examined in induced and uninduced BC3-shBcl2, BJAB-shBcl2 and their control cell lines, BC3-shC and BJAB-shC using propidium iodide (PI) staining and flow ctyometry analysis using previously published protocols from our lab [47], [52]. Briefly, 10^6^ cells were collected, washed, fixed, then treated with PI staining and RNase A digestion, followed by examination of cellular apoptotic levels. Each sample was analyzed six times. Statistical analysis was carried out using one-way ANOVA (Analysis of Variance).

## References

[1] Chang Y, Cesarman E, Pessin MS, Lee F, Culpepper J (1994). Identification of herpesvirus-like DNA sequences in AIDS-associated Kaposi's sarcoma.. Sicence.

[2] Cesarman E, Chang Y, Moore PS, Said JW, Knowles DM (1995). Kaposi's sarcoma-associated herpesvirus-like DNA sequences in AIDS-related body-cavity-based lymphomas.. N Engl J Med.

[3] Boshoff C, Schulz TF, Kennedy MM, Graham AK, Fisher C (1995). Kaposi's sarcoma-associated herpesvirus infects endothelial and spindle cells.. Nat Med.

[4] Soulier J, Grollet L, Oksenhendler E, Cacoub P, Cazals-Hatem D (1995). Kaposi's sarcoma-associated herpesvirus-like DNA sequences in multicentric Castleman's disease.. Blood.

[5] Ohtsuki Y, Iwata J, Furihata M, Takeuchi T, Sonobe H (1999). Ultrastructure of Kaposi's sarcoma-associated herpesvirus (KSHV)/human herpesvirus-8 (HHV-8) in a primary effusion lymphoma cell line treated with tetradecanoyl phorbol acetate (TPA).. Med Electron Microsc.

[6] Wang YC, Zhang Q, Montalvo EA (1998). Purification of Kaposi's sarcoma-associated herpesvirus (human herpesvirus 8) and analyses of the structural proteins.. J Virol Methods.

[7] Jenner RG, Albà MM, Boshoff C, Kellam P (2001). Kaposi's sarcoma-associated herpesvirus latent and lytic gene expression as revealed by DNA arrays.. J Virol.

[8] Sarid R, Flore O, Bohenzky RA, Chang Y, Moore PS (1998). Transcription mapping of the Kaposi's sarcoma-associated herpesvirus (human herpesvirus 8) genome in a body cavity-based lymphoma cell line (BC-1).. J Virol.

[9] Dittmer D, Lagunoff M, Renne R, Staskus K, Haase A (1998). A cluster of latently expressed genes in Kaposi's sarcoma-associated herpesvirus.. J Virol.

[10] Sharp TV, Wang HW, Koumi A, Hollyman D, Endo Y (2002). K15 protein of Kaposi's sarcoma-associated herpesvirus is latently expressed and binds to HAX-1, a protein with antiapoptotic function.. J Virol.

[11] Davis DA, Rinderknecht AS, Zoeteweij JP, Aoki Y, Read-Connole EL (2001). Hypoxia induces lytic replication of Kaposi sarcoma-associated herpesvirus.. Blood.

[12] Haque M, Davis DA, Wang V, Widmer I, Yarchoan R (2003). Kaposi's sarcoma-associated herpesvirus (human herpesvirus 8) contains hypoxia response elements: relevance to lytic induction by hypoxia.. J Virol.

[13] Cai Q, Lan K, Verma SC, Si H, Lin D (2006). Kaposi's sarcoma-associated herpesvirus latent protein LANA interacts with HIF-1 alpha to upregulate RTA expression during hypoxia: Latency control under low oxygen conditions.. J Virol.

[14] Miller G, Heston L, Grogan E, Gradoville L, Rigsby M (1997). Selective switch between latency and lytic replication of Kaposi's sarcoma herpesvirus and Epstein-Barr virus in dually infected body cavity lymphoma cells.. J Virol.

[15] Yu Y, Black JB, Goldsmith CS, Browning PJ, Bhalla K (1999). Induction of human herpesvirus-8 DNA replication and transcription by butyrate and TPA in BCBL-1 cells.. J Gen Virol.

[16] Bowser BS, DeWire SM, Damania B (2002). Transcriptional regulation of the K1 gene product of Kaposi's sarcoma-associated herpesvirus.. J Virol.

[17] Deng H, Song MJ, Chu JT, Sun R (2002). Transcriptional regulation of the interleukin-6 gene of human herpesvirus 8 (Kaposi's sarcoma-associated herpesvirus).. J Virol.

[18] Rimessi P, Bonaccrosi A, Stürzl M, Fabris M, Brocca-Cofano E (2001). Transcription pattern of human herpesvirus 8 open reading frame K3 in primary effusion lymphoma and Kaposi's sarcoma.. J Virol.

[19] Haque M, Chen J, Ueda K, Mori Y, Nakano K (2000). Identification and analysis of the K5 gene of Kaposi's sarcoma-associated herpesvirus.. J Virol.

[20] Seaman WT, Quinlivan EB (2003). Lytic switch protein (ORF50) response element in the Kaposi's sarcoma-associated herpesvirus K8 promoter is located within but does not require a palindromic structure.. Virology.

[21] Wang Y, Yuan Y (2007). Essential role of RBP-Jkappa in activation of the K8 delayed-early promoter of Kaposi's sarcoma-associated herpesvirus by ORF50/RTA.. Virology.

[22] Ueda K, Ishikawa K, Nishimura K, Sakakibara S, Do E (2002). Kaposi's sarcoma-associated herpesvirus (human herpesvirus 8) replication and transcription factor activates the K9 (vIRF) gene through two distinct cis elements by a non-DNA-binding mechanism.. J Virol.

[23] Song MJ, Deng H, Sun R (2003). Comparative study of regulation of RTA-responsive genes in Kaposi's sarcoma-associated herpesvirus/human herpesvirus 8.. J Virol.

[24] Liang Y, Ganem D (2004). RBP-J (CSL) is essential for activation of the K14/vGPCR promoter of Kaposi's sarcoma-associated herpesvirus by the lytic switch protein RTA.. J Virol.

[25] Wong EL, Damania B (2006). Transcriptional regulation of the Kaposi's sarcoma-associated herpesvirus K15 gene.. J Virol.

[26] Song MJ, Brown HJ, Wu TT, Sun R (2001). Transcription activation of polyadenylated nuclear rna by rta in human herpesvirus 8/Kaposi's sarcoma-associated herpesvirus.. J Virol.

[27] Song MJ, Li X, Brown HJ, Sun R (2002). Characterization of interactions between RTA and the promoter of polyadenylated nuclear RNA in Kaposi's sarcoma-associated herpesvirus/human herpesvirus 8.. J Virol.

[28] Masa SR, Lando R, Sarid R (2008). Transcriptional regulation of the open reading frame 35 encoded by Kaposi's sarcoma-associated herpesvirus.. Virology.

[29] Gonzalez CM, Wong EL, Bowser BS, Hong GK, Kenney S (2006). Identification and characterization of the Orf49 protein of Kaposi's sarcoma-associated herpesvirus.. J Virol.

[30] Sakakibara S, Ueda K, Chen J, Okuno T, Yamanishi K (2001). Octamer-binding sequence is a key element for the autoregulation of Kaposi's sarcoma-associated herpesvirus ORF50/Lyta gene expression.. J Virol.

[31] Deng H, Young A, Sun R (2000). Auto-activation of the rta gene of human herpesvirus-8/Kaposi's sarcoma-associated herpesvirus.. J Gen Virol.

[32] Byun H, Gwack Y, Hwang S, Choe J (2002). Kaposi's sarcoma-associated herpesvirus open reading frame (ORF) 50 transactivates K8 and ORF57 promoters via heterogeneous response elements.. Mol Cells.

[33] Wang SE, Wu FY, Yu Y, Hayward GS (2003). CCAAT/enhancer-binding protein-alpha is induced during the early stages of Kaposi's sarcoma-associated herpesvirus (KSHV) lytic cycle reactivation and together with the KSHV replication and transcription activator (RTA) cooperatively stimulates the viral RTA, MTA, and PAN promoters.. J Virol.

[34] Liu Y, Cao Y, Liang D, Gao Y, Xia T (2008). Kaposi's sarcoma-associated herpesvirus RTA activates the processivity factor ORF59 through interaction with RBP-Jkappa and a cis-acting RTA responsive element.. Virology.

[35] Green DR, Evan GI (2002). A matter of life and death.. Cancer Cell.

[36] Flanagan AM, Letai A (2008). BH3 domains define selective inhibitory interactions with BHRF-1 and KSHV BCL-2.. Cell Death Differ.

[37] Cory S, Adam JM (2002). The Bcl2 family: regulators of the cellular life-or-death switch.. Nat Rev Cancer.

[38] Chipuk JE, Moldoveanu T, Llambi F, Parsons MJ, Green DR (2010). The BCL-2 family reunion.. Mol Cell.

[39] Brychtová S, Brychta T, Kotrsová L, Pilka R, Tichý M (2000). Expression of Bcl-2 in dysplastic and neoplastic cervical lesions in relation to cell proliferation and HPV infection.. Neoplasma.

[40] Tsujimoto Y, Cossman J, Jaffe E, Croce CM (1985). Involvement of the bcl-2 gene in human follicular lymphoma.. Science.

[41] Yip KW, Reed JC (2008). Bcl-2 family proteins and cancer.. Oncogene.

[42] Higashiyama M, Doi O, Kodama K, Yokouchi H (1995). High prevalence of bcl-2 oncoprotein expression in small cell lung cancer.. Anticancer Res.

[43] Harada N, Hata H, Yoshida M, Soniki T, Nagasaki A (1998). Expression of Bcl-2 family of proteins in fresh myeloma cells.. Leukemia.

[44] Leiter U, Schmid RM, Kaskel P, Peter RU, Krähn G (2000). Antiapoptotic bcl-2 and bcl-xL in advanced malignant melanoma.. Arch Dermatol Res.

[45] Ohmori T, Podack ER, Nishio K, Takahashi M, Miyahara Y (1993). Apoptosis of lung cancer cells caused by some anti-cancer agents (MMC, CPT-11, ADM) is inhibited by bcl-2.. Biochem Biophys Res Commun.

[46] Harima Y, Harima K, Shikata N, Oka A, Ohnishi T (1998). Bax and Bcl-2 expressions predict response to radiotherapy in human cervical cancer.. J Cancer Res Clin Oncol.

[47] Bourgarel-Rey V, Savry A, Hua G, Carré M, Bressin C (2009). Transcriptional down-regulation of Bcl-2 by vinorelbine: identification of a novel binding site of p53 on Bcl-2 promoter.. Biochem Pharmacol.

[48] Wang XD, Ou TM, Lu YJ, Li Z, Xu Z (2010). Turning off transcription of the bcl-2 gene by stabilizing the bcl-2 promoter quadruplex with quindoline derivatives.. J Med Chem.

[49] Onyshchenko MI, Gaynutdinov TI, Englund EA, Appella DH, Neumann RD (2009). Stabilization of G-quadruplex in the BCL2 promoter region in double-stranded DNA by invading short PNAs.. Nucleic Acids Res.

[50] Breitschopf K, Haendeler J, Malchow P, Zeiher AM, Dimmeler S (2000). Posttranslational modification of Bcl-2 facilitates its proteasome-dependent degradation: molecular characterization of the involved signaling pathway.. Mol Cell Biol.

[51] Lan K, Kuppers DA, Verma SC, Sharma N, Murakami M (2005). Induction of Kaposi's sarcoma-associated herpesvirus latency-associated nuclear antigen by the lytic transactivator RTA: a novel mechanism for establishment of latency.. J Virol.

[52] Lu J, Verma SC, Murakami M, Cai Q, Kumar P (2009). Latency-associated nuclear antigen of Kaposi's sarcoma-associated herpesvirus (KSHV) upregulates survivin expression in KSHV-Associated B-lymphoma cells and contributes to their proliferation.. J Virol.

[53] He Z, Liu Y, Liang D, Wang Z, Robertson ES (2010). Cellular corepressor TLE2 inhibits replication-and-transcription- activator-mediated transactivation and lytic reactivation of Kaposi's sarcoma-associated herpesvirus.. J Virol.

[54] Junn HJ, Kim JY, Seol DW (2010). Effective knockdown of multiple target genes by expressing the single transcript harbouring multi-cistronic shRNAs.. Biochem Biophys Res Commun.

[55] Nakamura H, Lu M, Gwack Y, Souvlis J, Zeichner SL (2003). Global changes in Kaposi's sarcoma-associated virus gene expression patterns following expression of a tetracycline-inducible Rta transactivator.. J Virol.

[56] Lukac DM, Kirshner J, Ganem D (1999). Transcriptional activation by the product of open reading frame 50 of Kaposi's sarcoma-associated herpesvirus is required for lytic viral reactivation in B cells.. J Virol.

[57] Lukac DM, Renne R, Kirshner JR, Ganem D (1998). Reactivation of Kaposi's sarcoma-associated herpesvirus infection from latency by expression of the ORF 50 transactivator, a homolog of the EBV R protein.. Virology.

[58] Wen HJ, Minhas V, Wood C (2009). Identification and characterization of a new Kaposi's sarcoma-associated herpesvirus replication and transcription activator (RTA)-responsive element involved in RTA-mediated transactivation.. J Gen Virol.

[59] Ballestas ME, Chatis PA, Kaye KM (1999). Efficient persistence of extrachromosomal KSHV DNA mediated by latency-associated nuclear antigen.. Science.

[60] Cotter MA, Robertson ES (1999). The latency-associated nuclear antigen tethers the Kaposi's sarcoma-associated herpesvirus genome to host chromosomes in body cavity-based lymphoma cells.. Virology.

[61] Moore PS, Boshoff C, Weiss RA, Chang Y (1996). Molecular mimicry of human cytokine and cytokine response pathway genes by KSHV.. Science.

[62] Moore PS, Chang Y (2001). Molecular virology of Kaposi's sarcoma-associated herpesvirus.. Philos Trans R Soc Lond B Biol Sci.

[63] Wang S, Liu S, Wu MH, Geng Y, Wood C (2001). Identification of a cellular protein that interacts and synergizes with the RTA (ORF50) protein of Kaposi's sarcoma-associated herpesvirus in transcriptional activation.. J Virol.

[64] Yang Z, Wood C (2007). The transcriptional repressor K-RBP modulates RTA-mediated transactivation and lytic replication of Kaposi's sarcoma-associated herpesvirus.. J Virol.

[65] Wang SE, Wu FY, Fujimuro M, Zong J, Hayward SD (2003). Role of CCAAT/enhancer-binding protein alpha (C/EBPalpha) in activation of the Kaposi's sarcoma-associated herpesvirus (KSHV) lytic-cycle replication-associated protein (RAP) promoter in cooperation with the KSHV replication and transcription activator (RTA) and RAP.. J Virol.

[66] Galluzzi L, Brenner C, Morselli E, Touat Z, Kroemer G (2008). Viral control of mitochondrial apoptosis.. PLoS Pathog.

[67] Cam M, Handke W, Picard-Maureau M, Brune W (2010). Cytomegaloviruses inhibit Bak- and Bax-mediated apoptosis with two separate viral proteins.. Cell Death Differ.

[68] Everett H, McFadden G (1999). Apoptosis: an innate immune response to virus infection.. Trends Microbiol.

[69] Gruffat H, Sergeant A (1994). Characterization of the DNA-binding site repertoire for the Epstein-Barr virus transcription factor R.. Nucleic Acids Res.

[70] Chen LW, Chang PJ, Delecluse HJ, Miller G (2005). Marked variation in response of consensus binding elements for the Rta protein of Epstein-Barr virus.. J Virol.

[71] Young RL, Korsmeyer S (1993). A negative regulatory element in the bcl-2 5′-untranslated region inhibits expression from an upstream promoter.. Mol Cell Biol.

